# Supplementary material for: Genomic Profiling of Multidrug-Resistant Swine Escherichia coli and Clonal Relationship to Human Isolates in Peru
Source: Antibiotics (Basel). 2023 Dec 18;12(12):1748. doi: 10.3390/antibiotics12121748 (PMC10740509; doi:10.3390/antibiotics12121748)
Supplement: Supplementary file 1 [file antibiotics-12-01748-s001.zip › Figure S2.pdf]

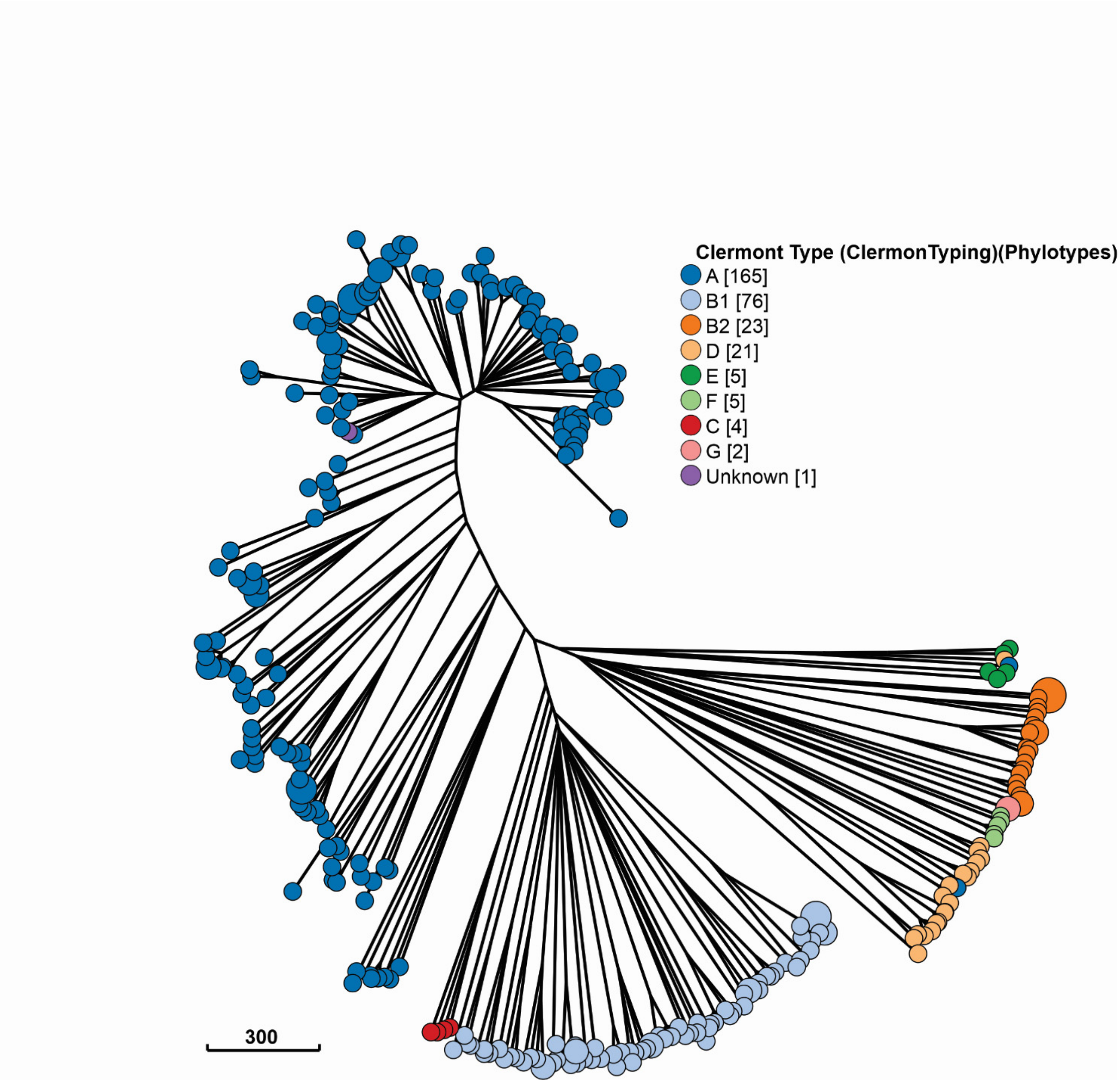

Figure S2. GrapeTree inferred using the Neighbor-joining (RapidNJ) algorithm based on the cgMLST V1 + Hierarchical Clustering (HierCC) V1 scheme of 302 Peruvian genomes according to the Clermont type. The numbers in brackets are the number of isolates belonging to a Clermont type.
